# A Nomogram for the Prediction of Cessation of Migraine Among Patients With Patent Foramen Ovale After Percutaneous Closure

**DOI:** 10.3389/fneur.2020.593074

**Published:** 2020-10-23

**Authors:** Enfa Zhao, Hang Xie, Yushun Zhang

**Affiliations:** Department of Structural Heart Disease, The First Affiliated Hospital of Xi'an Jiaotong University, Xi'an, China

**Keywords:** migraine, headache, patent foramen ovale closure, MIDAS, nomogram

## Abstract

**Objective:** This study aimed to develop and validate a nomogram to predict cessation of patent foramen ovale (PFO) patients with migraine headache after percutaneous closure.

**Methods:** A total of 247 eligible patients with PFO and migraine after percutaneous closure between May, 2016 and May, 2018 were divided into a development cohort (*n* = 149) and a validation cohort (*n* = 98). The primary end point was cessation of migraine at follow-up of 1 year after the procedure measured by the Migraine Disability Assessment Score (MIDAS). In the development cohort, the LASSO regression was used data dimension reduction. A multivariable logistic regression analysis was used to develop the predicting nomogram. The performance of the nomogram was assessed by concordance index (C-index), calibration and clinical usefulness. The results were validated in the validation cohort.

**Results:** Migraine with aura, history of antiplatelet, and the right-to-left shunt (RLS) at rest were identified as significant predictors based on the analysis of multivariate logistic regression. The nomogram incorporating these variables showed good calibration and discrimination in the development cohort with C-index of 0.906 (95% CI: 0.847–0.965), which was confirmed using the validation cohort with C-index of 0.827 (95% CI: 0.751–0.903). The nomogram showed good agreement between prediction by nomogram and actual observation. Furthermore, the decision curve indicated that the novel nomogram was clinically useful.

**Conclusion:** The novel nomogram showed favorable predictive accuracy for cessation of migraine among patients with PFO after percutaneous closure and might provide constructive guidance in clinical decision making.

## Introduction

Migraine is a complex neurological disorder that affects ~13% of the general population between the ages of 20 and 64 years with a higher prevalence observed in females and preceded by an aura in almost 36% of patients (1, 2). Migraine can pose a significantly detrimental effect on quality of life, with negative repercussions on work, social activities, and family life in healthy young adults (3). So far, its etiology and exact pathophysiology remain obscure (2, 4). Growing body of evidence reveals an association between mid to large RLS through the PFO and migraine (5–7). The foramen ovale is a flap-like communication in the atria septum secundum after birth, which is present in ~25% of adults (8). The underlying concept linking migraine and PFO is that migraine may be triggered by chemicals that reach the brain through PFO-RLS (9). The possible underlying mechanism is that the chemicals passed through the pulmonary circulation via a RLS without the metabolism in the lungs and entered the arterial circulation, which stimulates receptors in susceptible individuals in a concentration and causes the migraine phenomenon. Although the pathophysiology of migraine is not fully understood, the presence of PFO is believed to play a role in the pathophysiology. Prior nonrandomized observational studies have demonstrated that closure of PFO-RLS reduces the frequency and severity of migraine, particularly in patients with migraine with aura, and about 70–80% of patients who underwent PFO closure reported abolishment or improvement in their migraine attacks after PFO closure (5, 10–12). However, three randomized clinical trials of PFO closure for refractory migraine have failed to reach their primary endpoints (13–15). In the PREMIUM (Prospective, Randomized Investigation to Evaluate Incidence of Headache Reduction in Subjects with Migraine and PFO Using the AMPLATZER PFO Occluder to Medical Management) trial, complete migraine remission for 1 year occurred in 8.5% in the closure group (15). In the MIST (Migraine Intervention with STARFlex Technology) trial, the primary end point of migraine headache cessation in closure group was 4.05% at followed up for 6 months (13). Even at 5-year follow-up, 8.8% patients in the PFO closure group still presented with chronic migraine (16). The available evidence suggests that not all migraine patients with PFO benefit from such treatment. Therefore, in this study, we aimed to develop and validate a nomogram to predict cessation of migraine among patients with PFO after percutaneous closure.

## Methods and Materials

### Participants

This study was a part of the prospective registered clinical trial (https://register.clinicaltrials.gov/; NCT02777359). The study was approved by the ethics committee of the First Affiliated Hospital of Xi'an Jiaotong University, and all patients or their relatives provided written informed consent. Between May, 2016 and May 30, 2018, a total of 285 patients referred to the department of structural heart disease in our hospital for PFO-related migraine were included. Clinical history of migraine with or without aura was defined according to the International Headache Society criteria (17). The PFO-RLS was diagnosed using contrast transesophageal echocardiography (c-TEE) and contrast transesophageal echocardiography (TEE) examination, as described in our previous studies (18, 19). The grading of PFO-RLS was defined based on the following criteria: when no, 1–10 bubbles, 11–30 bubbles, and >30 bubbles (or left atrial opacity) were detected, the RLS was considered to be negative, mild, moderate, and extensive, respectively. All patients diagnosed with substantial RLS under Valsalva maneuver who underwent PFO closure were included. Migraineurs suffered ≥2 headache days/month averaged over the past 3 months and migraine without acute change or fluctuation were included. Besides, migraineurs with near-daily migraine were excluded from this study since they will tend to report a significant headache improvement even in the absence of migraine elimination. Patients with other cardiovascular defects, pulmonary arteriovenous malformation, the presence of intracardiac thrombi, bacteremia or active infections, active endocarditis, coagulopathy, platelet disorder, portal hypertension or, contraindication to aspirin or clopidogrel were excluded. Patients during pregnancy or with history of allergy or do not wish to participate were also excluded.

The clinical data were obtained via reviewing structured questionnaire recorded these patients' medical information. These data included patient age, gender, body mass index, history of hypertension status, hyperlipidemia, cerebral infarction, transient ischemic attack, history of diabetes mellitus, smoking status, history of preventive medications, history of antiplatelet treatment, migraine with or without aura, PFO with or without atrial septal aneurysm, PFO size, occluder size, type, red blood cell count, white blood cell count, platelets count, and PFO-RLS grading at rest. The validated Migraine Disability Assessment Score (MIDAS) was used to assess the incidence and severity of migraine headache before and after the PFO closure (20). In short, this five-point questionnaire evaluates the burden of migraine episodes in the past 3 months, their impact on working, social, leisure and family activities and the perceived pain.

### PFO Closure Procedure and Follow-Up

All procedures were performed with use of an Amplatzer® Septal Occluder (St. Jude Medical, Inc., St. Paul, Minn, USA) and Cardi-O-fix PFO Occluder (Starway Medical Technology, Beijing, China). The detailed surgical procedures were described in our previous study (21). After the procedure, all patients were treated with low-molecular-weight heparin at 10 U/(kg·h) for 48 h, aspirin 100 mg/day for 6 months, and clopidogrel 50–75 mg/day for 3 months following device implantation. All patients were follow-up every 3 months during the first year. All patients were followed at 3, 6, and 12 months after device implantation. During the follow-up, the MIDAS questionnaire was completed over telephone or in outpatients for each patient at each time point ± 5 days. Patients were asked to reexamine c-TTE to assess residual shunts if possible. The clinical endpoint is reached when the complete migraine remission event occurs according to the MIDAS at the follow-up at 1 year.

### Variables Selection and of the Nomogram Construction

All included patients who performed the PFO closure were divided into a development cohort and a validation cohort at a ratio of 3:2 using computer-generated random numbers. In practice, the least absolute shrinkage and selection operator (LASSO) model with an L1 penalty, which reduces the regression coefficient by applying a penalty proportional to its size from a large amount of candidates (22), was used to select the optimal predictive features in risk factors from the PFO patients with migraine after closure in the development cohort. Variables with nonzero coefficients in the LASSO regression were selected. Next, multivariable logistic regression analysis was used to construct a predicting model by incorporating the variable selected in the LASSO regression model. Backward step-wise selection was applied by using the likelihood ratio test with Akaike's information criterion as the stopping rule. A *P*-value for a variable <0.05 in analyses was considered to indicate statistical significance, and the variable was treated as a vital prognostic variable in prognostic prediction. The identified prognostic predictors were used to develop a predicting nomogram for complete migraine remission by using the development cohort.

### Assessment and Validation of the Nomogram

To quantify the discrimination performance of the novel nomogram, Harrell's concordance index (C-index) was measured (equivalent to the area under the ROC curve) (23). The C-index was used to assess the discrimination of the nomogram in both cohorts. Comparison of the C-index of a different model based on risk of paradoxical embolism (RoPE) was performed in two cohorts (24). Calibration plots were used to explore the performance of the nomogram. The calibration plot presented mean prediction probabilities on the x-axis and mean observed probabilities on the y-axis. A calibration plot along the 45° line would indicate a perfect calibration model between the bootstrap-predicted probabilities and the actual outcomes, any deviation above or below the 45° line suggests under or overestimation. Hosmer-Lemeshow statistic was also used to test the calibration performance in both cohorts, which tested the fitting precision between the observed and predicted outcome, and a significant test statistic indicates that the model does not calibrate perfectly (25).

### Clinical Utility

To explore the implications of the nomogram in clinical practice, we compared performance of the model to RoPE-based model using decision curve analysis (DCA) by quantifying the net benefits at different threshold probabilities in both cohorts (26). The method is based on the principle that the relative harms of false positives and false-negatives can be expressed in terms of a probability threshold. As a comprehensive method for evaluating and comparing different diagnostic and prognostic models, DCA can be used to explore whether the nomogram-assisted decisions would improve patient outcomes.

### Statistical Analyses

Continuous variables were expressed as mean and SD or median (interquartile range), and categorical variables were expressed as number and proportion as appropriate. The results were compared using the chi-squared test or Fisher's exact test. Continuous variables were compared using the *t*-test or Mann-Whitney U test for variables with an abnormal distribution. The Wilcoxon's rank-sum test for continuous variables and the chi-squared test for categorical variables were performed between two cohorts. We computed the AUC with a 95% confidence interval by using 1,000 bootstrap re-sampling. A nomogram was generated based on the multivariate prediction model using rms package in R software. ROC analysis was performed to calculate the area under curve (AUC) into evaluating the diagnostic performance of the models. All statistical tests were performed using R statistical software version 3.6.3. All statistical tests were two-tailed, and *P*-values < 0.05 were considered significant.

## Results

### Patient Clinical Characteristics

**Figure 1:**
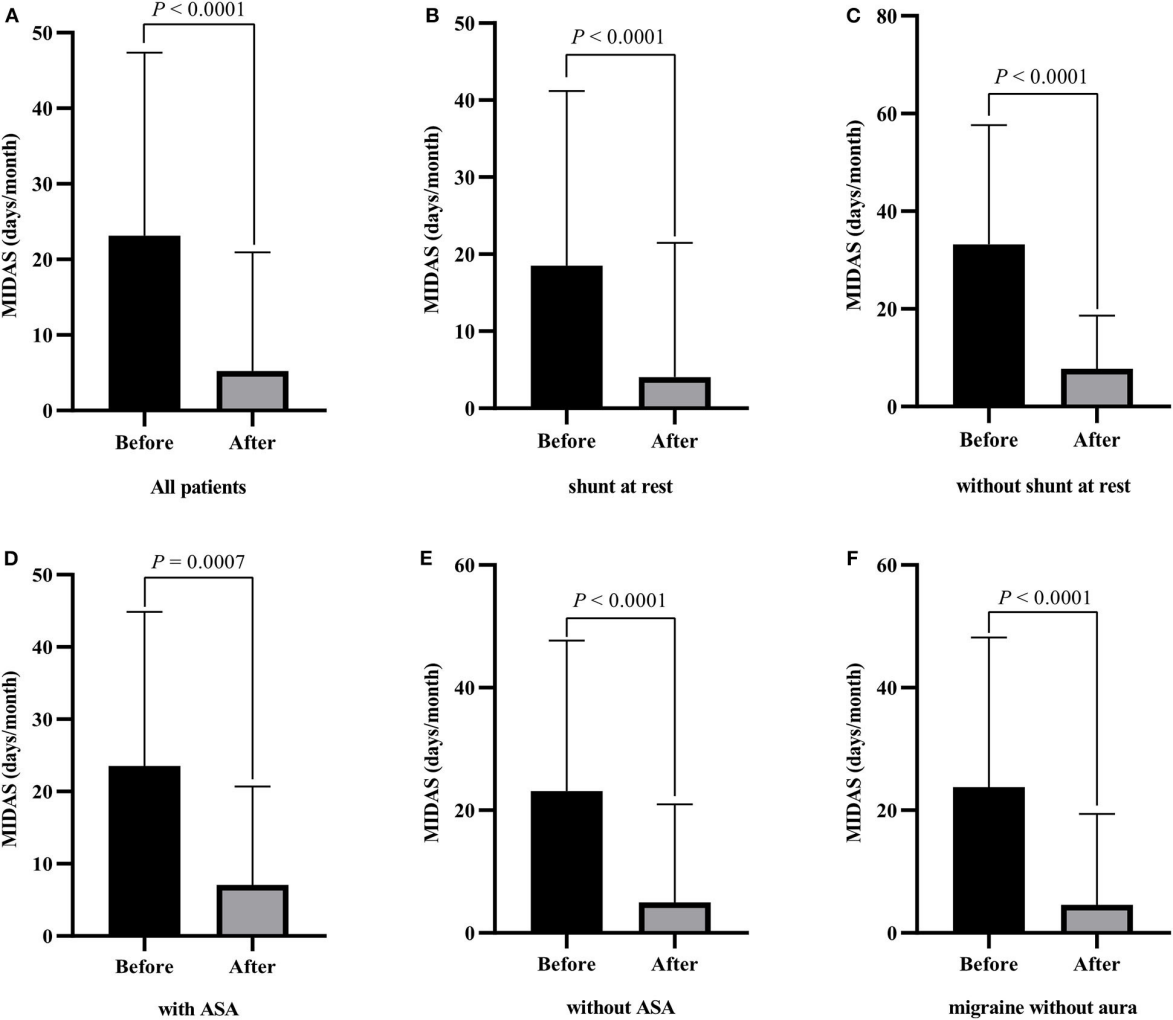
Changes of the migraine disability assessment score (MIDAS) at baseline and 1 year after PFO closure in patients with migraine. **(A)** all patients; **(B)** patients with shunt at rest; **(C)** patients without shunt at rest; **(D)** patients with ASA; **(E)** patients without ASA; **(F)** migraine without aura.

On the basis of all the inclusion criteria, a total of 247 patients underwent transcatheter PFO closure and completed the follow-up at 1 year. The procedure was successful in all patients, and all patients experienced symptomatic improvement with a mean MIDAS score of 23.15 at baseline and mean MIDAS score of 5.23 at 1 year follow-up (Figure 1A, *P* < 0.001). Subgroup analysis was performed to explore the probability of migraine cessation according to the different conditions, such as aura vs. no aura, ASA vs. no ASA, shunt at rest vs. no shunt at rest. As revealed in Figures 1B–F, migraineurs without aura, with ASA, without ASA, with shunt at rest, and without shunt at rest also experienced symptomatic improvement in terms of MIDAS score. In the development cohort, 91 (61.1%) patients with migraine eliminated, and 57 (58.1%) patients with migraine complete eliminated in the validation cohort. The clinical characteristics of patients with PFO and migraine in the two groups are shown in Table 1.

**Table 1:** Patient characteristic in the development and validation cohorts.

	**Development cohort (*N* = 149)**	**Validation cohort (*N* = 98)**	***P*-value**
Age (mean, ±SD)	37.39 ± 13.61	37.63 ± 13.67	0.8924
Sex (*n*, %)			0.753
Female	99 (66.4)	67 (68.3)	
Male	50 (33.6)	31 (31.7)	
BMI (kg/m^2^)	22.7 (20.3–25.6)	22.8 (19.9–24.6)	0.2454
Aura			0.4999
Yes	9 (6.0)	4 (4.1)	
No	140 (94.0)	94 (95.9)	
Atrial septal aneurysm (ASA)			0.2175
Yes	15 (10.1)	15 (15.3)	
No	134 (89.9)	83 (84.7)	
History of diabetes			0.102
Yes	4 (2.7)	0	
No	145 (97.3)	98 (100)	
History of hypertension			0.1983
Yes	11 (7.4)	12 (12.2)	
No	138 (92.6)	86 (87.8)	
History of hyperlipidemia			0.3637
Yes	4 (2.7)	1 (1.0)	
No	145 (97.3)	97 (99.0)	
History of smoking			0.5993
Yes	25 (16.8)	14 (14.3)	
No	124 (83.2)	84 (85.7)	
History of antiplatelet			0.0957
Yes	101 (67.8)	76 (77.6)	
No	48 (32.2)	22 (22.4)	
History of TIA			<0.001
Yes	139 (93.3)	5 (5.1)	
No	10 (6.7)	93 (94.9)	
History of cerebral infarction			0.476
Yes	11 (7.4)	5 (5.1)	
No	138 (92.6)	93 (94.9)	
RBC (x1,012/L)	4.44 (4.18–4.72)	4.49 (4.18–4.85)	0.2465
WBC (x109/L)	5.59 (4.87–6.78)	5.63 (4.75–6.93)	0.4144
PLT (x109/L)	203 (175–243)	218 (175–246.7)	0.7852
History of preventive medications			0.4559
Yes	95 (63.8)	67 (68.3)	
No	54 (36.2)	31 (31.7)	
PFO size			0.6235
>4 mm	7 (4.7)	6 (6.1)	
<4 mm	142 (95.3)	92 (93.9)	
Occluder size			0.6539
18/25 mm	99 (66.4)	60 (61.2)	
25/25 mm	27 (18.1)	18 (18.4)	
25/35 mm	9 (6.0)	10 (10.2)	
30/30 mm	14 (9.4)	10 (10.2)	
Occluder type			0.6088
Amplatzer	44 (29.5)	26 (26.5)	
Cardi-O-fix	105 (70.5)	72 (73.5)	
RLS at rest			0.1209
No	47 (31.5)	31 (31.6)	
Grade I	62 (41.6)	50 (5.2)	
Grade II	10 (6.7)	8 (8.1)	
Grade III	30 (20.1)	9 (9.1)	
Migraine cessation			0.6476
Yes	91 (61.1)	57 (58.1)	
No	58 (38.9)	41 (41.9)	
White matter lesions in MRI			0.9058
Yes	34 (22.8)	23 (23.5)	
No	115 (77.2)	75 (76.5)	

### Feature Selection and Nomogram Construction

**Figure 2:**
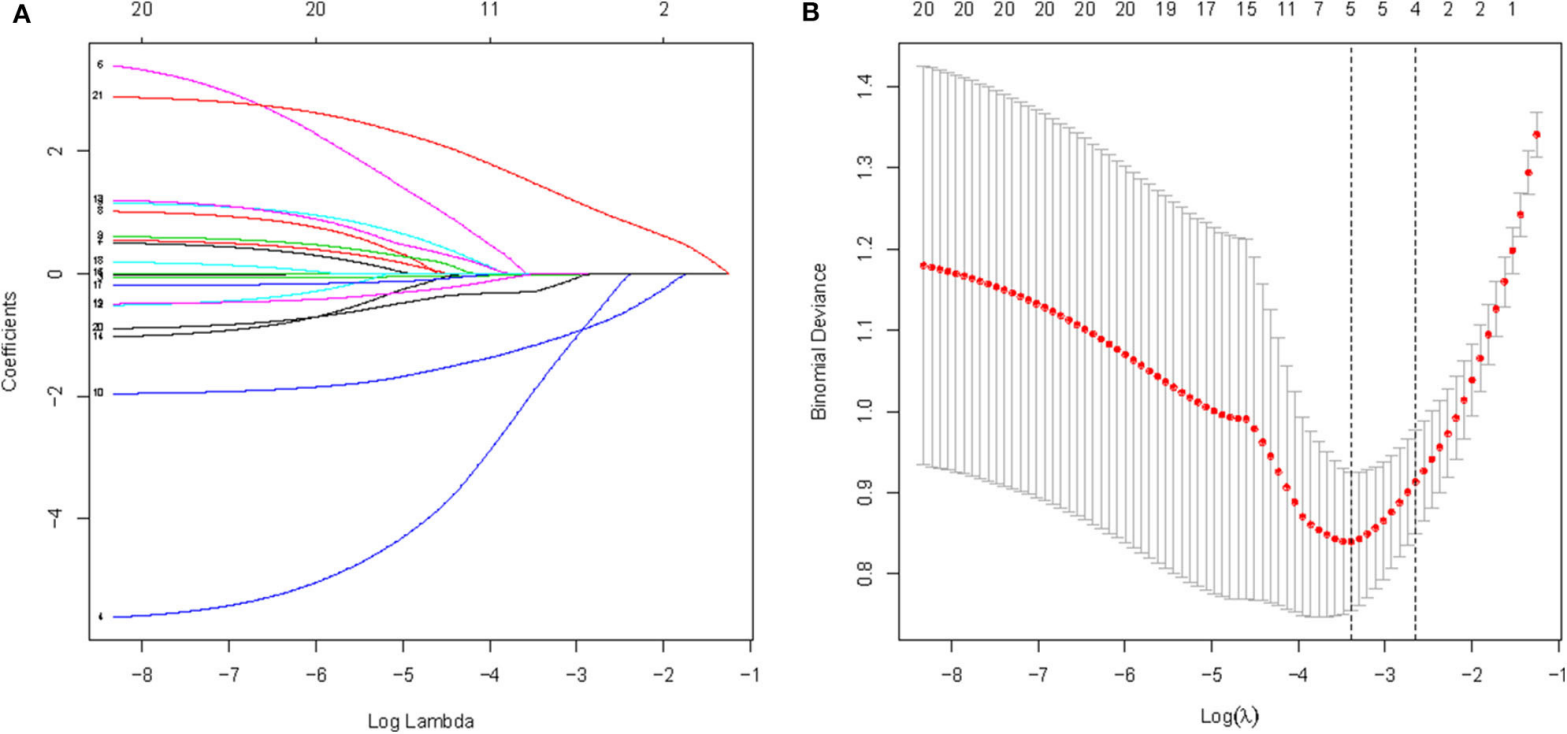
A logistic LASSO regression was used to select potential feature of cessation of migraine among candidate variables used 5-fold cross-validation. **(A)** LASSO coefficient profiles of the candidate features; **(B)** Optimal feature selection according to AUC value; LASSO, least absolute shrinkage and selection operator.

**Figure 3:**
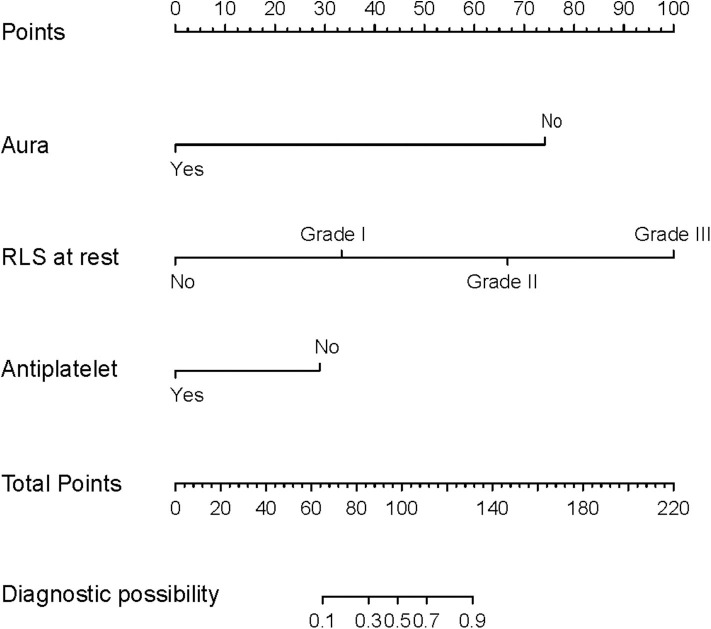
Nomogram predicting the probability of cessation of migraine at 1 year follow-up in patients with migraine after PFO closure. Using the variable of RLS at rest, a vertical line can be drawn from that variable to the points scale. After repeating the process for other variables, the scores for each variable can be summed and located on the “Total Points” axis. Finally, a vertical line can be drawn straight down from the plotted total point axis to the probability axis to locate the possibility of migraine cessation.

All available variables were fitted into the LASSO regression model. Variables with a regression coefficient nearly equal to 0 after the shrinkage process are excluded from the model, while variables with nonzero regression coefficient in the LASSO regression model are common strongly related to the outcome variable. As a result, BMI, occluder type, migraine with or without aura, history of antiplatelet, and RLS at rest with nonzero coefficients were identified (Figure 2). Multivariable logistic regression analysis began with the identified candidate variables. A logistic regression analysis identified the migraine with or without aura, history of antiplatelet, and RLS at rest as independent predictors (Table 2). The model that incorporated the above independent variables was used to develop a nomogram (Figure 3).

**Table 2:** Prediction factors for migraine cessation in PFO patients after closure at 1 year follow-up.

**Variables**	**Odds ratios**	**95% CI**	***P*-value**
BMI (kg/m^2^)	0.9319	0.8038–0.9903	0.26712
Occluder type	0.4448	0.1332–1.4006	0.1717
Migraine (with vs. without aura)	0.0061	0.00013–0.1349	**0.00386**
History of antiplatelet (Yes vs. No)	0.144	0.01924–0.6421	**0.02372**
RLS at rest	12.473	5.0101–36.3348	**<0.0001**

*Statically significant P-value given in bold, P < 0.05*.

### Calibration and Validation of the Nomogram

**Figure 4:**
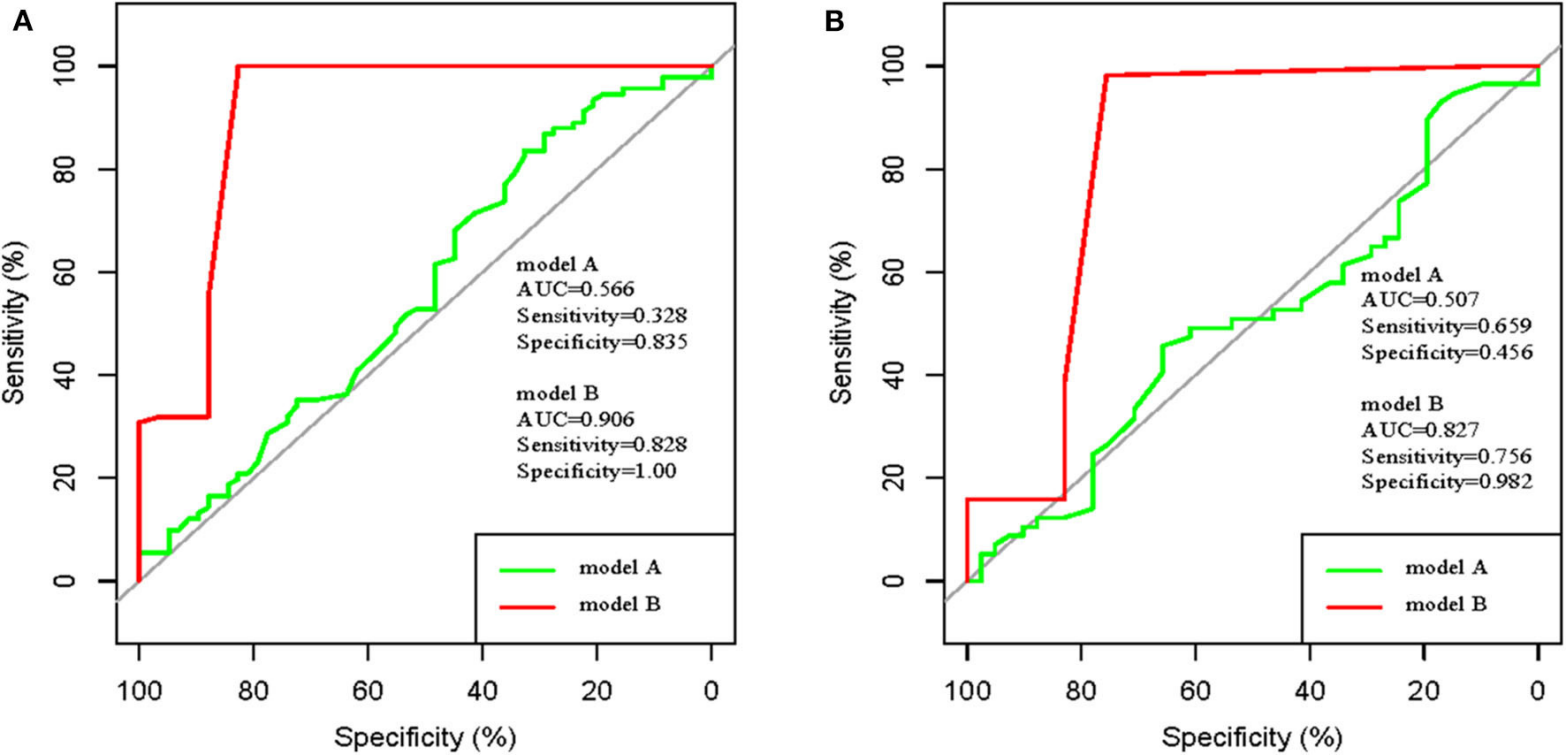
ROC curve of model A (RoPE-based model) and ROC curve of model B (our established model). **(A)** development cohort; **(B)** validation cohort.

The performance of novel nomogram was validated in the two cohorts, and was compared with the RoPE-based model. Discrimination was excellent for the novel model with AUC of 0.905 (95% CI: 0.847–0.965), sensitivity of 0.828, and specificity of 1 in the development cohort. The discriminative power is obviously higher than the RoPE-based model with AUC of 0.566 (Figure 4A). Additionally, the satisfactory discrimination was confirmed in the validation cohort with AUC of 0.827 (95% CI: 0.751–0.903), sensitivity of 0.756, and specificity of 0.982 (Figure 4B). The discrimination was still higher than the RoPE-based model with AUC of 0.507. Therefore, in the novel risk nomogram, apparent performance showed a good prediction capability.

**Figure 5:**
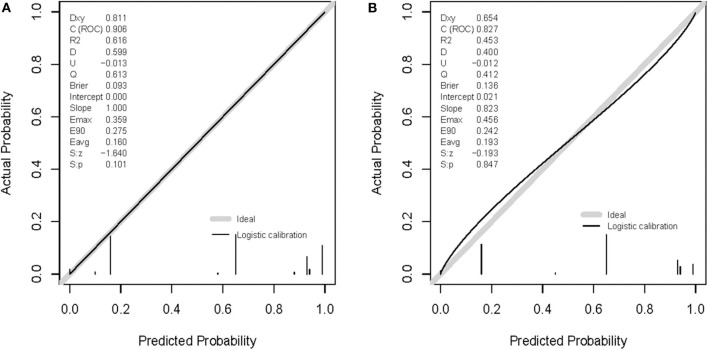
Calibration curves of the nomogram prediction. A calibration curve resulting from the logistic model shows good calibration when applied to the development **(A)** and validation **(B)** cohorts.

The shape of the slope on the calibration plots demonstrate that the novel nomogram is well calibrated in the development cohort (Figure 5A). The Hosmer and Lemeshow statistical test for the observed data for the nomogram supported the goodness-of-fit of the model (*P* = 0.101) in the development cohort. Furthermore, the Hosmer-Lemeshow goodness-of-fit test yielded a nonsignificant statistic (*P* = 0.847) in the validation cohort (Figure 5B), which demonstrated that there was no departure from perfect fit. Therefore, the novel nomogram performed well using both the development and validation cohorts.

### Clinical Usefulness of the Nomogram

**Figure 6:**
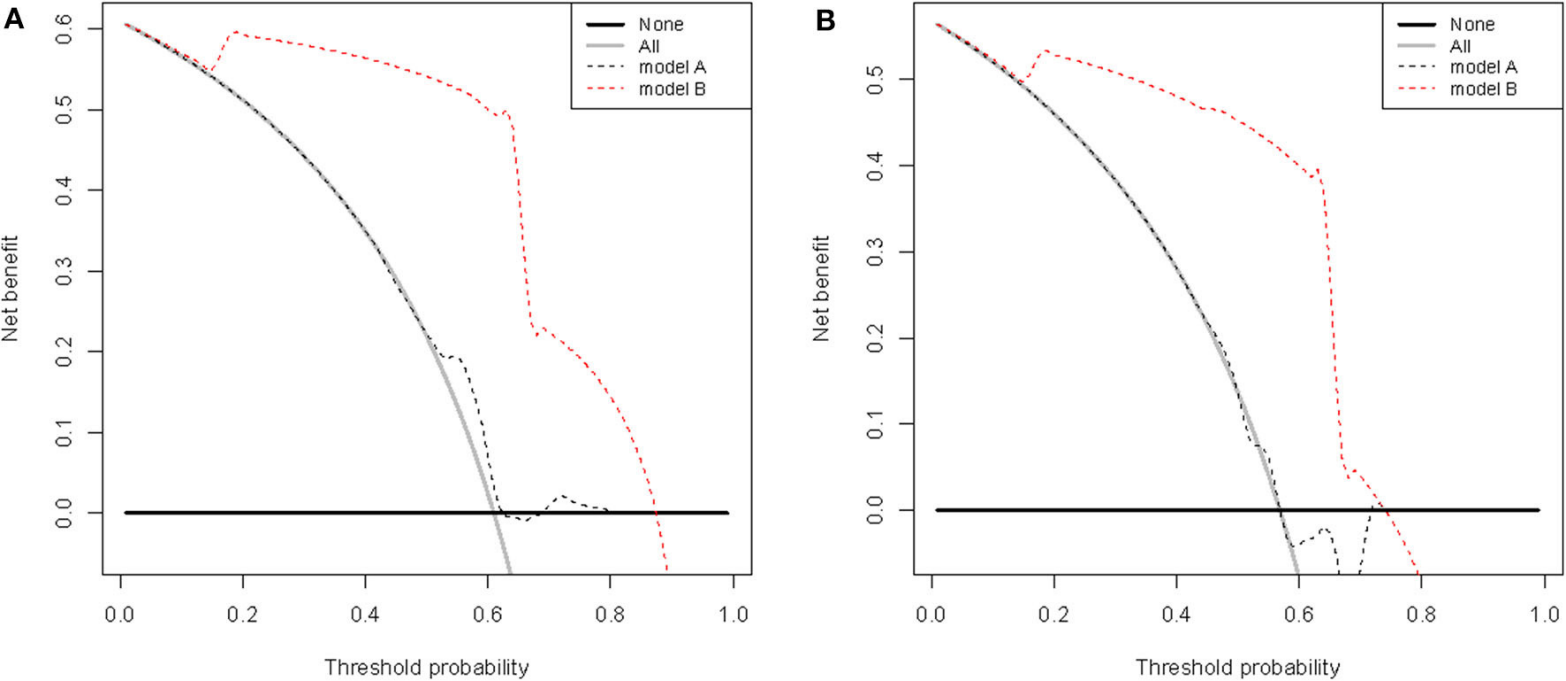
Decision curve analysis of RoPE-based model (model A) and nomogram (model B) in terms of elimination of migraine in the development **(A)** and validation **(B)** cohorts. The x-axis indicates the threshold probability, and the y-axis demonstrates the net benefit.

The clinical usefulness of nomogram was estimated using decision curve analysis (DCA) by quantifying the net benefits for a range of threshold probabilities. The DCA for the established nomogram and that for the model with RoPE integrated is presented in Figures 6A,B. The novel model showed a higher net benefit with a broader range of threshold probability. The decision curve showed that using the nomogram to predict elimination of migraine among patients with PFO after percutaneous closure adds more benefit than either the treat-all-patients strategy or the treat-none strategy, which indicated the novel nomogram to be clinical useful.

## Discussion

Migraine is among the most common neurological disorder and has been acknowledged as one of the most important causes of disability burden that often significantly affect patients' lives (27). Migraine and the presence of a PFO have been associated with each other, although the exact pathophysiological mechanisms linking migraine headache and PFO remain unclear (28). Observational studies reveal that some improvement of migraine would be expected if the PFO were to be closed. The extent of improvement or elimination of migraine headache attack symptoms was variable. There are other studies reporting the useless PFO closure in migraine with and without aura. A prospective study of 80 PFO patients with migraine presented a significant benefit with PFO closure at 2-year follow up, with complete relief in 96.8% of the patients with migraine aura, and significant improvement of migraine in 87.5% of all patients with migraine regardless of with or without aura (29). In a retrospective cohort study, 150 patients suffered from migraine, including 96 patients with migraine with aura, were subjected to PFO closure. After a median follow-up of 4.9 years, among migraine with aura, headaches disappeared in 39% and improved in 49%; while among migraine without aura, headaches disappeared in 26% and improved by at least 50% in 42% patients (30). A recent meta-analysis included eight studies demonstrated a significant improvement of migraine in 81% of migraine with aura patients compared to only 63% of migraine without aura patients (31). According to 20 observational studies that explored the effect of PFO closure on migraine, the elimination of migraine was reported in 10–83% of patients, improvement in 14–83%, and no change in 1–54% patients (32). All three trials failed to meet their primary end points defined as migraine cessation and >50% reduction in migraine days at 1 year (33). Therefore, identifying factors affecting remission and constructing a nomogram is important to predict prognosis of migraine patients with PFO undergoing percutaneous closure.

Recent years, nomograms are commonly used as prognostic tools in oncology and medicine. Nomograms provide improved individual prediction accuracy of future clinical outcome by combining the effects of various variables associated with these events to aid better clinical decision making (34). The present study was the first to apply a nomogram to predict cessation of migraine among patients with PFO after percutaneous closure. In this study, we developed and validated a novel relatively accurate prediction tool for cessation of migraine among patients with PFO after percutaneous closure using a prospective cohort study. The nomogram incorporates three items of the migraine with or without aura, history of antiplatelet, and RLS at rest. Incorporating these easily available factors into an easy-to-use nomogram facilitates the PFO individualized prediction of migraine elimination after percutaneous closure. The nomogram performed well in predicting outcome, and its prediction was supported by the C-index (0.906 and 0.827 for the development and validation cohorts, respectively) and the calibration curve. When compared with the RoPE-based model, the nomogram demonstrated better predictive accuracy for cessation of migraine. Furthermore, the DCA showed that using the nomogram to predict elimination of migraine among patients with PFO after percutaneous closure brings more benefit than the RoPE-based model.

In this study, three significant predictors (migraine with or without aura, RLS at rest, and history of antiplatelet) were identified using a multivariable logistic regression analysis model. There was an obvious difference between the presence of PFO in migraine with aura and migraine without aura. Migraine with aura cessation ranged from 28.6 to 92.3%, whereas migraine without aura elimination varied from 13.6 to 82.9%. Migraine with aura improvement ranged from 4.17 to 64.3%, and migraine without aura improvement varied from 0 to 68.2% (28). PFO closure benefit migraine patients with aura and show better results and aura may be reduced, which demonstrated in observational and RCT studies (33, 35). As we known, 5-hydroxytryptamine (5HT) has an important role in migraine, through high 5HT venous concentrations as a result of platelet activation, and drugs that act on 5HT receptors are widely used to treat migraine. A randomized study in the UK demonstrated that aspirin, which has an antiplatelet action, weakened migraine compared with controls (36). Migraine with aura after PFO closure in the first few weeks is terminated if the patient is prescribed clopidogrel 75 mg/d for the first 4 weeks after the closure procedure (37). A previous study revealed that MIDAS scores among the migraine patients with moderate or large PFO-RLS were notably higher than those in patients from the mild PFO-RLS group and non-RLS group (*P* < 0.05) (38).

A history of antiplatelet also found contributed to the relief of migraine. For example, a previous study had reported clopidogrel, a thienopyridine platelet P2Y12 receptor inhibitor, could reduce migraine headache symptoms in certain patients with PFO, which demonstrating a platelet-based mechanism/trigger (39). Clopidogrel may have a primary prophylactic role in migraine patients, and could contribute to select patients who would benefit from PFO closure (40). A recent study further confirmed that clopidogrel could use as an effective complementary prophylactic for PFO migraineurs with poor response to routine prophylactics (41). Furthermore, transcatheter PFO closure was effective at improving moderate and severe migraine symptoms in patients with large RLS shunt as well as the elimination of aura (42). All these evidence helps to explain the role of the identified predictors in the model.

Although this is the first nomogram to predict cessation of migraine among patients with PFO after percutaneous closure, the limitations should be considered. First, the sample size is rather small. However, this is the first study developing and validating a nomogram in migraine patients undergoing PFO closure. In addition to being a possible component in medication overuse/chronic migraine, antiplatelet therapy with salicylate may also be a confounder regarding the outcome as all patients were given this after intervention and salicylate daily may be expected to affect migraine either through its antimigraine effect. Besides, it is a single-center analysis, and a multicenter validation study should be performed to confirm the performance of the novel model in future studies.

## Conclusion

We developed and validated a nomogram incorporating migraine with or without aura, history of antiplatelet, and RLS at rest to predict elimination of migraine among patients with PFO after percutaneous closure in a Chinese population. The novel nomogram showed favorable performance and might provide constructive guidance in clinical decision making.

## Data Availability Statement

The raw data supporting the conclusions of this article will be made available by the authors, without undue reservation.

## Ethics Statement

The study was approved by the ethics committee of the First Affiliated Hospital of Xi'an Jiaotong University, and all patients or their relatives provided written informed consent.

## Author Contributions

EZ and HX is the principle investigator, conducted statistical analysis and draft the manuscript. EZ performed data management and bioinformatics analysis. EZ, HX, and YZ edited and revised the manuscript. All authors contributed to the article and approved the submitted version.

## Conflict of Interest

The authors declare that the research was conducted in the absence of any commercial or financial relationships that could be construed as a potential conflict of interest.
